# Solution NMR Determination of the CDHR3 Rhinovirus-C Binding Domain, EC1

**DOI:** 10.3390/v13020159

**Published:** 2021-01-22

**Authors:** Woonghee Lee, Ronnie O. Frederick, Marco Tonelli, Ann C. Palmenberg

**Affiliations:** 1 Department of Chemistry, University of Colorado Denver, Denver, CO 80204, USA; woonghee.lee@ucdenver.edu; 2 Department of Biochemistry, National Magnetic Resonance Facility at Madison, University of Wisconsin-Madison, Madison, WI 53706, USA; rofrederick@wisc.edu (R.O.F.); tonelli@nmrfam.wisc.edu (M.T.); 3 Department of Biochemistry, Institute for Molecular Virology, University of Wisconsin-Madison, Madison, WI 53706, USA

**Keywords:** rhinovirus C, receptor, CDHR3, NMR structure

## Abstract

Cadherin Related Family Member 3 (CDHR3) is the identified and required cellular receptor for all virus isolates in the rhinovirus-C species (RV-C). Cryo-EM determinations recently resolved the atomic structure of RV-C15a, and subsequently, a complex of this virus bound to CDHR3 extracellular domain 1 (EC1), the N-terminal portion of this receptor responsible for virus interactions. The EC1 binds to a hypervariable sequence footprint on the virus surface, near the 3-fold axis of icosahedral symmetry. The key contacts involve discontinuous residues from 3 viral proteins, VP1, VP2 and VP3. That single cryo-EM EC1 structure, however, could not resolve whether the virus-receptor interface was structurally adaptable to accommodate multiple virus sequences. We now report the solution NMR determination of CDHR3 EC1, showing that this protein, in fact, is mostly inflexible, particularly in the virus-binding face. The new, higher resolution dataset identifies 3 cis-Pro residues in important loop regions, where they can influence both rigidity and overall protein conformation. The data also provide clarification about the residues involved in essential calcium ion binding, and a potential CDHR3 surface groove feature that may be involved in native protein interactions with cellular partners.

## 1. Introduction

Receptor-mediated endocytosis, triggered by virus binding to appropriate cells, is a key entry step in the initiation of rhinovirus infections. The majority of rhinovirus-A (RV-A) and rhinovirus-B (RV-B) genotypes (~100 “major” group) require the ubiquitous, albeit transiently expressed intracellular adhesion molecule-1 (ICAM-1) as their cellular receptor, defining ciliated pulmonary epithelial cells marked with this protein, as suitable hosts. A “minor” group of RV-A genotypes (~10×) can also trigger entry through interactions with low-density lipoprotein receptors (LDLR), although it has never been definitively proven whether this is a true alternative pathway or a preferred parallel backup mechanism used by this cohort, in the absence or downregulation of ICAM-1 (reviewed in [1]).

In contrast to either pathway, genotypes (57×) of the more recently described rhinovirus-C (RV-C) have an alternative requirement for CDHR3 (Cadherin Related Family Member 3), a constitutively-expressed airway epithelium protein restricted to the apical surfaces of ciliated cells [2,3]. The first clinical description of this gene came from genetic screenings implicating CDHR3 as a key correlate in children with the most severe forms of asthma and found that a single nucleotide polymorphism (rs6967330, “G” vs. “A”) encoding a Cys529 to Tyr529 substitution (C529Y) changes the protein density on cell surfaces [4]. Almost simultaneously with this work, parallel screens with susceptible and non-susceptible tissue culture samples identified CDHR3 as the required RV-C cellular receptor [2]. Transformed or transduced cultured cells became significantly more susceptible to virus when the higher-density Y529 protein was expressed, relative to the lower-density C529. It is now clear the ancestral, albeit lower frequency “A” allele of this gene (Y529) is among the strongest known genetic correlates for childhood virus-induced asthma-susceptibility. The increased surface display by the Y529 protein creates a propensity in these children for more frequent and severe RV-C infections which trigger or fuel the ensuing critical asthma exacerbations [3].

Our continuing work on this system is aimed at a molecular understanding of the RV-C receptor interactions at the critical core of this process. The CDHR3 sequence predicts a rod-like arrangement of six linear tandem-repeat extracellular domains (EC), each of about 110 residues, preceded by a signal sequence (residues 1–19), and followed by a transmembrane domain (residues 714–734) and cytoplasmic tail (to residue 885). Initial structure models [5] suggested each EC segment assumed a canonical cadherin β-sandwich fold, packing 7 β-strands into 2 β-sheets (AGFC, BED), with occasional intervening short helical loops. Individual EC units include (roughly) residues 20–130, 138–227, 238–336, 347–452, 458–558 and 567–679. Classic cadherins are similar, but have five, not six stacked EC units [6]. Native CDHR3 also has three mapped N-linked glycosylation sites (EC2 N186, EC4 N384, EC6 N624) [7]. Residue 529 at the junction of the EC5+6 repeats is ideally located to help regulate adjacent stabilizing inter-domain Ca^++^ binding, without which CDHR3 unfolds, and is apparently unable to traffic properly from the ER to the Golgi [7]. NMR and biochemistry experiments are currently ongoing to confirm whether indeed, a Ca^++^ differential is the specific cause of the observed, crucial, allele-dependent phenotypes.

Only the N-terminal EC1 repeat (residues 20–126) has a central role in RV-C binding, in a Ca^++^-dependent, glycosylation-independent manner [7]. Bacterially-produced EC1 or EC1+2, properly refolded in the presence of Ca^++^ recapitulate virus-receptor interactions in vitro and can directly inhibit RV-C infection of susceptible cells for several viral genotypes. The respective structures of these recombinant materials, as bound to RV-C15a, were recently resolved by cryo-EM techniques to 3.1 Å and 3.4 Å resolution [8]. As expected, each protein bound through its EC1 domain, laying the flat AGF strands of the AGFC sheet, in tight proximity to loops presented by the VP1, VP2 and VP3 virus proteins, near the three-fold axis of icosahedral virus symmetry. Three monomers of EC1 saturated each symmetry axis when the density data were processed by icosahedral averaging, but for the larger EC1+2, and by extrapolation, for the full 6-domain CDHR3 receptor, steric inference imposed by the extended EC2 units would prevent more than one native protein from binding per three-fold vertex.

The AGF binding face places 20 EC1 residues’ side-chain or backbone atoms within 4 Å of viral VP1, VP2 and VP3 atoms, fitting with overall geometric complementarity into a shallow virion surface groove. Saturation mutagenesis of EC1 followed by virion binding tests, showed surprisingly, that only a handful of these receptor residues were collectively important to multiple RV-C genotypes. These included EC1 D102 (βF), P26 (βA*-A), and I23 (βA*) which interact, respectively, with VP2 R234, VP3 N56 and VP1 I276, each of which is a highly conserved aligned site among the known RV-C genotypes, but not the RV-A or RV-B. Various additional potential interactions in the remainder of the EC1 face, also proved important in that they could be affected by mutation, but only in a RV-C genotype-specific manner. For example, RV-C02 accepted EC1 H21G/Q, L24D, T28G, N99A and II00A mutations, all of which were conversely lethal to RV-C45 interactions. On the other hand, RV-C02 was sensitive to an EC1 Q104A disruption, tolerated by RV-C15, RV-C41 and RV-C45. Such different patterns of peripheral requirements held true for each of the four tested viruses, involving collectively, about half of the full cohort of putative EC1 binding contacts. Except for those few discontinuous, conserved virus residues noted above, each RV-C genotype presents a highly variable set of surrounding sequences to its receptor binding face. This means EC1 needs to complement somehow with a fit acceptable to each RV-C genotype. Our cryo-EM co-structure only sampled a single virus-bound EC1 conformation. We now report the analogous NMR solution structure of free EC1 for comparison.

## 2. Materials and Methods

### 2.1. Protein Production

Cloned expression of a functional human CDHR3 EC1 virus-binding domain has been described [7] along with the protein numbering system (GenBank IC58018) and domain delineation, originally predicted from a structure model [2,5] then confirmed by cryo-EM determination when complexed with virus [8]. Recombinant EC1 for NMR was produced by the following workflow. CDHR3 residues L20-P130 were engineered into a pET11a T7 promoter-based plasmid system, which adds a C-terminal hexa-histidine tag to the target protein, and relies on an *E. coli* Rosetta2(DE3)-pLysS host strain that efficiently translates potentially rare eukaryotic codons. A single colony of freshly transformed cells was transferred into medium (1 mL, 2xYT, plus ampicillin, 100 μg/mL and chloramphenicol, 35 μg/mL), before incubation for 3–4 h at 37 °C. MDAG medium (25 mL, defined minimal medium) [9] was added with incubation continued overnight (25 °C, shaking at 250 rpm). This inoculum was added to double-labeled M9 growth media (1 L M9 with 4 g of ^13^C-glucose and 1 g of ^15^NH_4_Cl, supplemented with appropriate antibiotics) with further growth at 37 °C for (typically) 4 h, until an optical density of 1.4 (600 nm) was reached. Protein overproduction was induced by isopropyl-β-D-thioglactopyranoside (IPTG, to 0.4 mM) and further culturing for 18 h at 25 °C. Cell harvest was by low speed centrifugation (4402× *g*, 20 min, 4 °C). The paste was re-suspended in lysis buffer (50 mL, Tris pH 8, 0.5 M NaCl, 0.2–0.8% nonyl phenoxypolyethoxylethanol detergent [NP40], 1 mM Tris-2-carboxyethyl phosphine [TCEP] or β-mercaptoethanol, 1 mM phenylmethylsulphonate fluoride [PMSF] serine protease inhibitor, 0.05% azide), and then ultrasonicated (15 min total, 1 s on, 4 s off, 50–60% power setting) with a Misonix ultrasonicator 3000. The sample was clarified by centrifugation (15,000× *g*, 30 min) retaining the inclusion-body pellet, which was washed with water and then lysis buffer (1% triton X-100), and recollected in between by centrifugation (15,000× *g*, 20 min). The final pelleted material was dissolved in 6 M guanidine, then dialyzed and refolded overnight against IMAC-A (50 mM Tris pH 8, 0.5 M NaCl, 5 mM imidazole, 0.05% azide, 5 mM CaCl_2_), clarified by centrifugation, then loaded onto IMAC resin (10 mL, Immobilized Metal Affinity Chromatography column, Qiagen, 1 mL/min flow rate). The column was washed with IMAC-A, then IMAC-A with 30 mM imidazole (5–10 column volumes), and then IMAC-A with 0.50 M imidazole (30 mL), which eluted the protein. Purity was checked by visualization of Coomassie-stained SDS-PAGE. After refolding and IMAC purification the protein was concentrated to 2 mL volume by centrifugation with a spin concentrator (Millipore filtration with molecular weight cut-off, 3.5 kDa) then loaded onto a gel filtration column (Superdex 75, HiLoad 16/60), before the final product was dialyzed against HKC buffer (50 mM HEPES pH 7, 300 mM KCl, 10 mM CaCl_2_), and reanalyzed by SDS-PAGE. The final samples were flash frozen in LN_2_.

### 2.2. NMR Sample Preparation

Before data collection, fresh EC1 materials, labeled with ^15^N or ^13^C/^15^N, were exchanged into HKC buffer (with 0.05% sodium azide and 10% D_2_O, Sigma-Aldrich, St. Louis, MO, USA) to a final concentration of ~0.4 mM. The samples (300 µL) were enclosed in 5-mm susceptibility-matched Shigemi NMR tubes (Shigemi, Allison Park, PA, USA).

### 2.3. NMR Data Collection

NMR data were acquired on Varian VNMRS (Agilent Technologies, Santa Clara, CA, USA) or Bruker Avance III spectrometers operating at 600 and 800 MHz (^1^H), equipped with cryogenic triple-resonance probes. The sample temperature was maintained at 293 K for all experiments. To assign sequence-specific backbone resonances, 2D ^1^H,^15^N-HSQC and 3D HNCACB, 3D CBCA(CO)NH, 3D HNCA, 3D HN(CO)CA, 3D HN(CA)CO and 3D HNCO spectra were collected. For the full assignment of the aliphatic ^1^H and ^13^C resonances, 2D constant-time ^1^H,^13^C-HSQC and 3D HBHA(CO)NH, 3D H(C)CH-COSY, 3D H(C)CH-TOCSY and 3D (H)CCH-TOCSY spectra were also acquired. Before the final structure calculation, additional 3D NOESY ^15^N-HSQC and 3D NOESY ^13^C-HSQC data sets were collected, using (for both) a 150 millisecond mixing time. The acquisition parameters of all 3D spectra are reported in Table S1. Each dataset was recorded using Non-Uniform Sampling (NUS) with rates ranging from 32% and 49% (with the higher sampling rate for NOESY spectra). Spectra were processed using NMRPipe [10] protocols, although those recorded with NUS, were reconstructed and processed using the SMILE [11] package.

### 2.4. Chemical Shift Assignment

The Wisconsin–Madison NMR facility has adopted an Integrative NMR Platform for analyzing data [12]. This includes the (semi-)automation tools of NMRFAM-SPARKY for assigning backbone and sidechain chemical shifts [13]. The backbone shifts, notably, peaks from 2D ^1^H,^15^N-HSQC, HNCO, HN(CA)CO, CBCA(CO)NH and HNCACB were initially picked by the APES plugin (two-letter-code *ae*) [14] and then verified by strip plots. CA/CB peaks from CBCA(CO)NH and HNCACB were loaded on one strip plot (two-letter-code *sp*) and CO peaks from HNCO and HN(CA)CO on another strip plot (two-letter-code *SP*). Returned, inspected peaks were input into I-PINE by the PINE-SPARKY.2 plugin (two-letter-code *ep*) [15,16] to conduct automated backbone chemical shift assignments. These were then verified by PINE-SPARKY plugins, PINE Graph (two-letter-code *pp)* and PINE Assigner (two-letter-code *pr*) [17].

For the sidechain assignments, the “predict-and-confirm” method was used. The *Transfer-and-Simulate* plugin (two-letter-code *ta*) created initial peak assignments, with additional “_s” tags on HBHA(CO)NH spectra, aided by PACSY database predictions [18]. Interactively peak positions were adjusted and confirmed with center-and-untag “cu”, untag “ut”, and merge pseudo-nomenclature “mt” tools to best match with the contour lines. Established sidechain assignments were mapped onto ^1^H,^13^C-HSQC to fix stereospecificity (two-letter-code *hc*) and then H(C)CH-TOCSY and (H)CCH-TOCSY were used to finalize the assignments.

### 2.5. Xaa-Pro Peptide Bond Conformations

Because the proline content of EC1 is relatively high (10 of 126 residues), the I-PINE web server flagged several possibilities for unusual backbone chemical shifts. Therefore, the *Xaa*-*Pro* peptide bond conformations were further evaluated with the proline conformation prediction tool in NMRFAM-SPARKY (two-letter-code *dp*) and also a Promega program [19] which looks specifically for C^β^ and C^γ^ chemical shifts as well-known indicators for *cis*-Prolines.

### 2.6. Protein Three-Dimensional Structure Determination

Assembly of the final three-dimensional structure used the PONDEROSA-C/S suite, coupled with Xplor-NIH algorithms [14,20]. Once the initial fold was calculated by the PONDEROSA automation plugin in NMRFAM-SPARKY (two-letter-code *c3*), automatically picking NOE cross peaks from 3D NOESY-^1^H,^15^N-HSQC and 3D NOESY-^1^H,^13^C-HSQC, the output was submitted to Ponderosa for analysis by the AUDANA algorithm [21]. Acquired distance and angle restraints were visually validated and then refined using Ponderosa Analyzer, the Ponderosa Connector plugin in NMRFAM-SPARKY (two-letter-code *up*), and also PyMOL (Schroedinger, LLC, New York, USA). After a few iterative passes using these parameters, calculations of structures with refined restraints by *Constraints only-X* option, which conducts high-temperature dynamics, simulated annealing and low-temperature dynamics, were evaluated with the PONDEROSA-C/S suite. The Xplor-NIH options [22] identified the lowest energy structure, the distance and dihedral angle restraints, and also identified three tightly-bound calcium ions. From among 400 calculated structures based on the lowest energy criteria, 20 were chosen and refined via a TIP3 water box using the *WaterRefineTools* module of the Xplor-NIH package. PSVS 1.5 was used to evaluate the overall structural quality of these states [23], including bound ions and water. The final EC1 structure ensemble was deposited (and validated by) in the Worldwide Protein Data Bank [24] with PDB ID: 7KNV. The associated chemical shifts data were deposited in BioMagResBank with accession number: 30812.

### 2.7. CDHR3 Model Building

Cryo-EM-determined coordinates (PDB ID 6ppo, 6psf) and density maps for EC1 and EC1+2 (partial), as bound to the RV-C15a virus have been published [8], as has a full 6-domain model for CDHR3, build with I-Tasser procedures [5]. Using the common EC2 domain for orientation, a new 6-domain model built with PyMol (Schroedinger, LLC) captured and linked virus-bound EC1+2 from the determined cryo-EM structure, to the EC4-6 domains, as previously modeled. For locale comparison, three N-linked glycosylation moieties (from PDB ID 5szn), were included proximal to Asn186 (EC2), Asn384 (EC4), and Asn624 (EC6). These native sites have been identified by mass spectrometry [7].

## 3. Results

### 3.1. Chemical Shift Assignments

**Figure 1:**
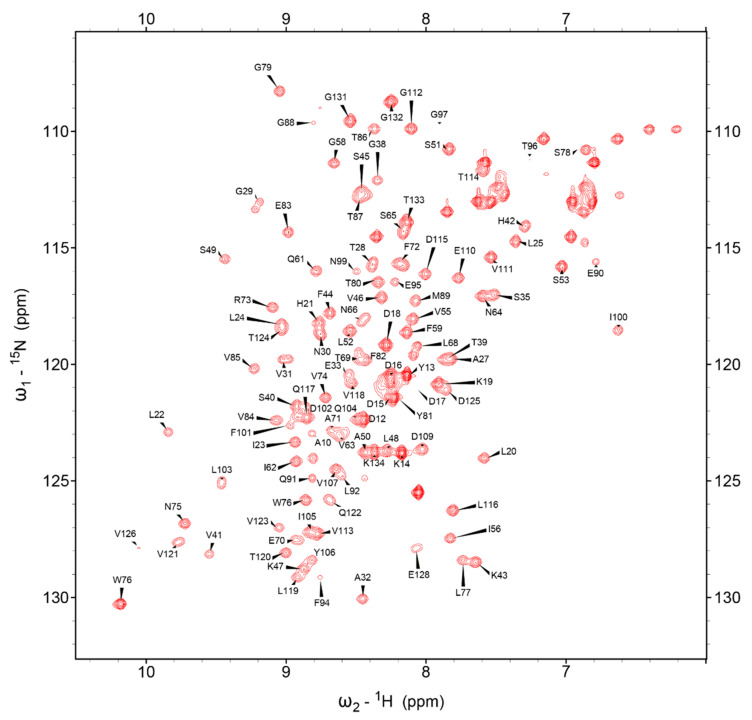
^1^H,^15^N-HSQC spectrum of EC1 with assignment labels.

Using the collective NMR spectra and described Methods, 516 of 528 (97.73%) backbone resonances (N, H, C^o^, C^α^, C^β^) could be assigned, excluding only the prolyl nitrogens. The resolved protein spans CDHR3 L20 to P130, omitting the recombinant N- and C-terminal cloning tags. The ^1^H,^15^N HSQC assignment map in Figure 1, shows excellent peak separation. However, conformational variability caused doublets or elongated signals for some residues (e.g., G29, V31, N66, L68, F72, V121 and Q122, see Supplementary Materials Figure S1). To resolve this, triple resonance experiments were referenced and compared before deciding on the major peak positions. The combined backbone and aliphatic sidechain assignments were 96.66% (1101 of 1139 atoms) for the L20-P130 region, a cohort more than strong enough to proceed to a full structure calculation.

### 3.2. Cis-Proline Analysis

During inspection of collected spectra, the NMRFAM-SPARKY peptidyl-prolyl cis-trans prediction tools reported three potential cis peptide bonds (L25-P26, F59-P60, N66-P67) with probabilities of 1.00, 1.00 and 0.98, respectively. The Promega analyses tools concurred in reporting these same bonds as potential cis conformations, with probabilities of 1.00, 1.00 and 0.96, respectively. Table 1 shows assigned C^β^ and C^γ^ chemical shifts of P26, P60 and P67 from the NMR spectra. Generally, differences larger than 8 ppm between ^13^C^β^ and ^13^C^γ^ chemical shifts (i.e., Table 1, column 4) indicate cis configurations, while difference values of 5 ppm or less are expected for trans peptidyl-prolyl bonds. This rotamer information was included in the Ponderosa Client program for automated NOE assignments, in the fold-calculation steps, and also when using the Xplor-NIH program to determine calcium ion binding at later stages of modeling. The final 20 structure models (described below) have ω dihedral angles between L25-P26, F59-P60 and N66-P67 at ~0°, typical values for cis Xaa-Pro peptidyl-prolyl bonds.

### 3.3. Structural Content Determined by Chemical Shift

**Figure 2:**
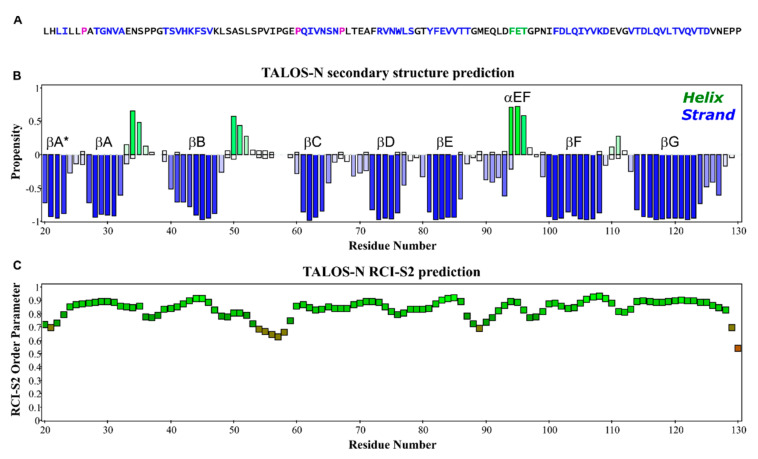
Structure content of EC1. (**A**) The primary sequence of EC1, L20-P130, is color-coded by STRIDE according to the determined EC1 solution structure (blue β-strand; magenta cis-Pro). (**B**) Secondary structure elements predicted by TALOS-N program (blue β-strand; green α-helix). (**C**) Backbone flexibility estimated by the RCI-S^2^ method.

The I-PINE web server gave complete secondary structure and flexibility predictions from assigned chemical shifts, as integrated by the TALOS-N program suite [25]. Confirming the previous cryo-EM results, Figure 2 shows the NMR determined structural content of EC1 is essentially all beta-strand elements (Figure 2A,B) with a short 3^10^ helix between F94-T96, and two partial helical turns between N34-S35 and A50-L52. The random coil index order (RCI-S^2^) [26] suggests on the whole, EC1 is quite rigid throughout its full sequence with values for only a few residue (P54, V55, I56, P57, G58, P129, P130) dipping below thresholds of 0.7 (Figure 2C). Only the C’ most residues, (P129, P130) have lower values. In a full-length CDHR3 context, this region functions as part of the linker to the EC2 domain.

### 3.4. Statistical Validity of Final Models

**Figure 3:**
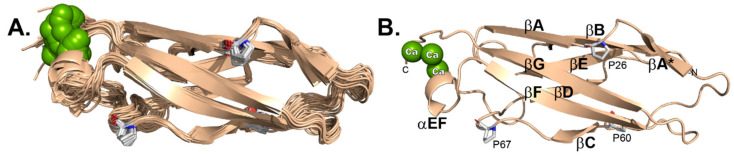
EC1 by NMR. (**A**) 20 models. (**B**) Lowest energy model (#1) labelled by structure features including cis-prolyl orientations.

The 20 selected 3D structures (Figure 3A) were finalized according to the parameters described in Table S2. A total 2018 distance constraints (short: 1085; medium: 133; long: 800) were used to define the structure, along with 25 hydrogen bond constraints and 211 dihedral angle constraints (Φ: 101; Ψ: 110) as derived from TALOS-N analysis. The most representative structure (#1, Figure 3B), of lowest energy, was picked by STRIDE analyses [27], after considering multiple, superimposed, selected regions (residues: 22–23, 30–31, 39–46, 62–65, 72–78, 81–86, 94–96, 100–108, 114–123). RMSD (root mean square deviation) values for the ensemble of 20 structures, were 0.68 Å and 1.36 Å for backbone versus all heavy atoms, respectively. MolProbity analysis [28] was 99.2% for “most favored”, 0.8% for “allowed” and none for “disallowed” with 16.05 clashscore. There were no consistently violated constraints identified by this structure calculation. The final XPLOR-NIH pseudopotential energy was 4743.01 kJ/mol.

### 3.5. Bound Ca++ Ions

**Figure 4:**
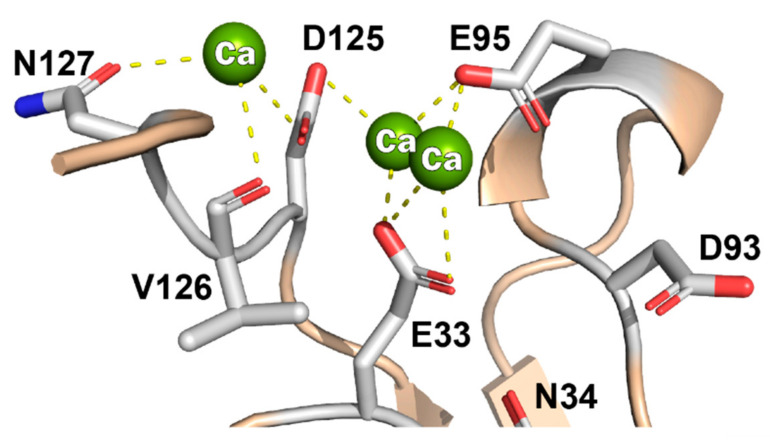
Bound Ca++ ions show PyMol-defined polar contacts in model #1.

Included in the dataset were unambiguous signals for 3 tightly bound Ca^++^ ions, localized near the C-terminus (Figure 4) at what would be the EC1–EC2 junction. These are stabilized by at least 3 polar contacts each, from the sidechains of E33 in the βAB loop, E95 in the βEF 3^10^ helix, D125 and N127, from the terminal tail, and the backbone carbonyl of V126. The sidechains of N34 of D93, which were predicted to interact from the cryo-EM determination are not in close contact with the ions in this higher resolution NMR structure.

### 3.6. Comparison of Solution-Determined and Virus-Bound EC1

**Figure 5:**
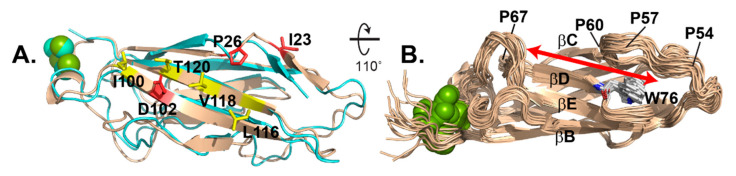
Structure comparison. (**A**) NMR model #10 (wheat) overlaid with cryo-EM determination (6ppo, cyan) highlights key virus binding residues (red) and others responsive to particular virus genotypes (yellow). (**B**) 20 models show a narrow, rigid backside groove (red arrow) anchored by W76.

The NMR suite fit well with the previously determined EC1 coordinates as bound to the RV-C15a virus (Figure 5A). The comparative RMSD for each state relative to the virus-bound structure varied from 1.486 Å (#10) to 2.138 Å (#5) with a median of 1.814 Å. Most of the flexibility manifest in loop regions, with the sheets, particularly on the virus binding face, being almost invariant (Figure 3A). Included on this face are I23, cis-P26 and D102, which according to mutagenesis are required for all tested virus interactions, and also I100, L116, V118, and T120, which were mutationally responsive to some viruses, but not others [8]. The NMR models also validate a shallow groove, 6–8 Å wide, between the βC-βD strands, anchoring the respective sheets. The groove is not part of the virus binding face. It is capped by W76 at one end of the βD strand, the only Trp moiety in the N-terminal half of CDHR3 (Figure 5B). The C-terminal portion of the βBC loop overhangs W76 forming an edge to the groove. This loop segment is entirely hydrophobic (PVIPGFP), stiffened by 3 Pro residues, the last of which is cis-Pro60. The groove itself extends ~30 Å, the entire length of EC1 with the far end, away from W76 defined by a sharp turn in the βCD loop, at cis-Pro67.

## 4. Discussion

The cryo-EM coordinates of native RV-C15a virus [29], when compared to those bound by CDHR3 receptor fragment EC1 [8], showed almost no evidence of induced fit on that virus, which might have helped mold these proteins together when they interacted. That finding was a little surprising, in that the EC1-virus footprint covers perhaps ~900 Å^2^ on each entity, and for the virus, includes almost the entirety of the predicted discontinuous NIM3 (Neutralizing Immunogen Site 3), of highly variable sequence. The 57 genotypes of RV-C each use this receptor but among their analogous contact faces, there is very poor conservation except for VP2 K/R234 (100%), VP3 N56 (100%) and VP1 I/V/L276 (97%). When these VP2 and VP3 residues were mutated in a RV-C15 context, the resulting virus was unable to interact with EC1 [8]. These are difficult experiments because the defective virus can only be isolated after RNA transfections. Reciprocal experiments, testing recombinant EC1 sequences, were more extensive because panels of mutated proteins could be readily isolated and compared. Against four different RV-C genotypes, only three EC1 residues in the AGC face could not be mutated with impunity. Among these, D102 in βF makes direct sidechain contact with VP2 K/R234 and with VP3 T204 in a surface region deemed “patch1”. EC1 changes to D102A/Q prevented binding by any RV-C.

The same was true for P26A changes within surface “patch3” encompassing the hydrophobic stretch included in βA* near the N-terminus. This whole region, LILLPA (L22-A27), has interactions with the virus hydrophobic VP1 tail, primarily I276. In addition to being non-polar, the cryo-EM structure shows the EC1 Pro26 backbone carbonyl in close contact with the ND2 of VP3 N56. Mutagenesis of either residue prevented all binding. Higher resolution NMR now classifies this interaction as probably dependent on the cis/trans status of Pro26. As observed experimentally [7], substituted (trans) amino acids would reorient βA*-βA, altering a good portion of the virus binding face. To better define the interactions of this and the other two cis residues, we will now try to rebuild the cryo-EM coordinates, forcing all determined cis-Pro into their alternate rotamers.

Before resolving EC1 by NMR, there was an expectation that the virus binding face might be partially flexible, able to accommodate multiple virus interface sequences. The previous mutagenesis experiments suggested that individual virus genotypes responded to different EC1 mutations, outside of the core, key residues. The sensitive responses spanned L116, Q117, V118 and T120 (marked yellow in Figure 5A), along the terminal βG strand, yet central to the AGF binding face. However, NMR does not identify very much flexibility here, or major shifts in these residues from the cryo-EM structure, meaning virus binding does not significantly deform EC1 on this face. Instead, NMR-identified doublets or elongated peaks indicating possible structural flexibility (e.g., G29, V31, N66, L68, F72, V121 and Q122, see Supplementary Materials Figure S1) localize to the back side of the protein, near cisPro67 and the Ca^++^ ions. For the most part, virus-binding must be a hard-dock interaction. Except for possible immunogenic variation in the involved VP3 surface loops (i.e., NIM3), each virus apparently accommodates the relatively fixed structure of EC1, with individual sequence changes, but not overt conformational ones.

No doubt, this is also the reason virus interactions are highly sensitive to EC1 Ca^++^ binding. If recombinant protein is not synthesized, refolded and maintained in a calcium buffer, it loses the ability to bind virus. Simple dilution into a non-calcium buffer has the same effect and is not reverted by subsequent re-addition [7]. Therefore, at least one of these ions is diffusible, perhaps the one coordinated by the more disordered C-terminal tail, D125, V126 and N127. Any disruption at this end of the protein would reorient the 3^10^ helix and its attached βF strand, displaying key residue, D102. Future NMR experiments with Cd^++^ substitutions are planned for EC1 and EC1+2, as well as the surface display EC5 + 6 units to better describe the distribution and requirements for these inter-domain stabilizing ions.

When natively displayed on cells, CDHR3, like other classic cadherins, is believed to accept binding partners for homotypic or heterotypic functional interactions. Although recombinant EC1 and EC1+2 are stable monomers, EC1-3 or larger iterations are difficult to maintain in solution [7]. It is currently unclear whether this is due to aggregation, misfolding, or multimerization. Classical cadherin dimerization is frequently mediated by EC1 strand swapping, reliant on terminal Trp exchanges [30,31]. In cells, mutation of CDHR3 W76, the only Trp in EC1-3, actually enhances RV-C binding [7], suggesting the virus preferentially recognizes receptor monomers. To date though, we have not identified the presumed missing interaction partner, whether it be a more terminal EC domain, or completely different protein. However, the EC1 βC-βD groove, opposite the virus binding face, is the probable locale for such an interaction. NMR now shows, two of the three cis-Pro residues help define this groove configuration and its long, hydrophobic surface profile (Figure 5B), displaying W76 for ready binding access. Given the current NMR higher resolution of these required surface elements, it should now be possible to “fish” in Y529-expressing pulmonary cell extracts for preferred CDHR3 partners that might fit this site.

**Figure 6:**
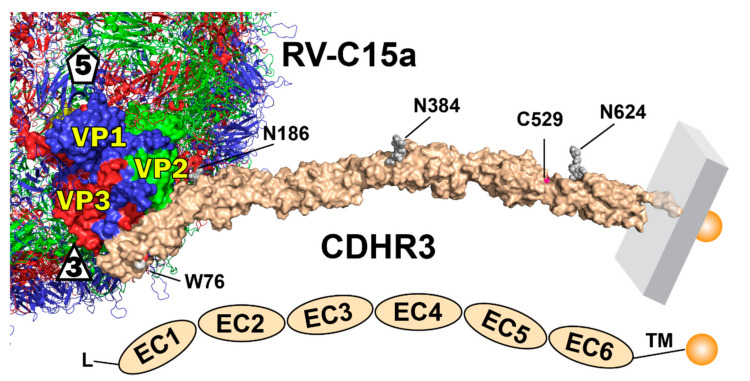
Current model for CDHR3 and its interaction with RV-C (see Section 2).

The collective structural and biochemical information makes it timely to create an updated model for CDHR3 in its full orientation and scale, when docked to virus (Figure 6).

In 2015, before real coordinates for an RV-C or CDHR3 were available, homology modeling [2] correctly predicted a linear arrangement of 6x linked EC units, but anticipated virus-receptor interactions via β-sheets from both EC1+2, across the virus two-fold axis of symmetry. At that time, this orientation seemed favorable because it spanned most of the probable RV-C conserved surface residues, left W76 surface-exposed, and evoked a putative role for EC2 N186, a known glycosylated residue that seemed contributory. The actual cryo-EM determinations [8,29], subsequently showed the computational model had correctly predicted a portion of the real footprint, but critically, EC1 the only required contact domain, actually bound at the three-fold axis, ~45° to the 2-fold virus axis, not perpendicular to it. The real orientation pushes the remainder of CDHR3 away from the virus and away from the five-fold symmetry axis [8]. Each native three-fold can only accommodate one CDHR3 moiety, not a symmetrical dimer or other symmetrical multimers. The model-only suggestions involving possible EC2 contacts, were consistent with reports that N186A and R166A mutations were inhibitory [7]. Later it was found those specific effects were fully or partially reversible if recombinant proteins were properly refolded [7]. Interestingly though, the cryo-EM structure siting EC1+2 onto RV-C15a, do now place N186 and R166 close to each other on the EC2 face proximal to the virus, possibly contributing some stability in cells, but not to mandatory recombinant interactions. In the future, perhaps a higher resolution cryo-EM structure, or solution NMR determination of the linked EC1+2, would be informative in better defining the exact orientation and functions of these residues. We are working on such preparations in parallel with materials for EC5+6 that encode both C529 and Y529 variations. Hopefully these proposed structures also will help resolve conformational differences that may influence intracellular CDHR3 trafficking, and their subsequent, all important surface display density.

## Figures and Tables

**Table 1:** ^13^C^β^ and ^13^C^γ^ chemical shifts of P26, P60 and P67 indicate *cis* Xaa-Pro peptidyl-prolyl bonds.

Residue	δ^13^C^β^ (ppm)	δ^13^C^γ^ (ppm)	Δδ(^13^C^β^–^13^C^γ^) (ppm)	ω Dihedral Angles in the 20 Selected Models (°)
P26	36.00	24.70	11.30	−1.98 (±0.51)
P60	35.45	24.88	10.57	−1.01 (±0.64)
P67	34.37	25.83	8.54	−0.45 (±0.47)

## Data Availability

The final EC1 structure ensemble was deposited in the Worldwide Protein Data Bank with PDB ID: 7KNV. The associated chemical shifts data were deposited in BioMagResBank with accession number: 30812.

## Supplementary Materials

The following are available online at https://www.mdpi.com/1999-4915/13/2/159/s1: Table S1: Experimental details for 3D spectra collected for backbone, side-chains assignments and structure calculation, Table S2: NMR structure statistics, Figure S1: Conformational variability.
