# Supplementary material for: Solution NMR Determination of the CDHR3 Rhinovirus-C Binding Domain, EC1
Source: Viruses. 2021 Jan 22;13(2):159. doi: 10.3390/v13020159 (PMC7911512; doi:10.3390/v13020159)
Supplement: Supplementary file 1 [file viruses-13-00159-s001.pdf]

Solution NMR Determination of the CDHR3 Rhinovirus-C Binding Domain, EC1

Woonghee Lee [1], Ronnie O. Frederick [2], Marco Tonelli [3], and Ann C. Palmenberg [4*]

Supplemental Material

Table S1. Experimental details for 3D spectra collected for backbone, side-chains assignments and structure calculation.[a]

Experiment	# Scans	Spectral Window (ppm) $^{1}H \times {}^{13}C(^{1}H^{\#}) \times {}^{15}N(^{13}C^{\&})$	Complex Points $^{1}H \times {}^{13}C(^{1}H^{\#}) \times {}^{15}N(^{13}C^{\&})$	Offset (ppm) $^{1}H \times {}^{13}C(^{1}H^{\#}) \times {}^{15}N(^{13}C^{\&})$	NUS sampling rate (%)
HNCACB	128	16×66.3×27	1024×64×32	4.82, 45.3, 119.1	32
CBCA(CO)NH	128	16×66.3×27	1024×60×30	4.82, 45.3, 119.1	32
HNCA	48	16×26.5×27	1024×64×39	4.82, 54.0, 119.1	36
HN(CO)CA	48	16×26.5×27	1024×64×39	4.82, 54.0, 119.1	36
HN(CA)CO	128	20×13.9×28	1024×40×32	4.82, 175.6, 119.3	49
HNCO	32	20×13.9×28	1024×40×32	4.82, 175.6, 119.3	49
HBHA(CO)NH	128	$20\times6.7^{\#}\times28$	$1024\times40^{\#}\times32$	$4.82, 4.82^{\#}, 119.3$	49
H(C)CH-TOCSY	32	$20\times8^{\#}\times69.6^{\&}$	$1024\times64^{\#}\times64^{\&}$	$4.82, 4.82^{\#}, 41.6^{\&}$	36
(H)CCH-TOCSY*	32	$16.3\times72.1\times72.1^{\&}$	$1024\times64\times64^{\&}$	$4.82, 42.2, 42.2^{\&}$	36
H(C)CH-COSY*	64	$16.3\times7.5^{\#}\times72.1^{\&}$	$1024\times48^{\#}\times48^{\&}$	$4.82, 4.82^{\#}, 42.2^{\&}$	36
NOESY ^{15}N-HSQC	32	$20\times13.3^{\#}\times28$	$1024\times72^{\#}\times38$	$4.82, 4.82^{\#}, 119.3$	49
NOESY ^{13}C-HSQC	32	$20\times12.1^{\#}\times58.3^{\&}$	$1024\times64^{\#}\times64^{\&}$	$4.82, 4.82^{\#}, 41.6^{\&}$	49

[a]All spectra were collected with a cryogenic probe at 600 or 800* MHz (^{1}H) and at 293 K.

Table S2. NMR structure statistics. Structural quality was evaluated by PSVS 1.5 for the selected residues † by STRIDE.

†

Parameter	Value
**NOE derived distance constraints**	
Short range ($\|i-j\| \le 1$)	1085
Medium range ($1 < \|i-j\| < 5$)	133
Long range ($\|i-j\| \ge 5$)	800
**Total**	2018
**Hydrogen bond constraints**	25
**Dihedral angle constraints**	
Φ	101
Ψ	110
**Total**	211
**Average root mean square deviations against lowest energy model coordinates (Å)†**	
Backbone atoms (N, Cα, C, O)	0.68±0.16
All heavy atoms	1.36±0.22
**PROCHECK *Z* scores (φ and ψ/all)**	-0.90/-1.24
**Ramachandran plot summary from MolProbity†**	
Most favored	99.2
Allowed	0.8
Disallowed	0
**MolProbity Clashscore (raw score / Z-score)**	16.05 / -1.23
**Root mean square deviations from ideal geometry**	
For bond lengths (Å)	0.015
For angles (°)	1.3
**XPLOR-NIH pseudopotential *E*(kJ/mol)**	4743.01±32.65
**Consistent violations (>30%)**	
Distance constraints (>0.5 Å)	0
Dihedral angle constraints (>5°)	0
van der Waals constraints (>0.2 Å)	0

Selected residues by STRIDE: 22, 23, 30, 31, 39-46, 62-65, 72-78, 81-86, 94-96, 100-108, 114-123.

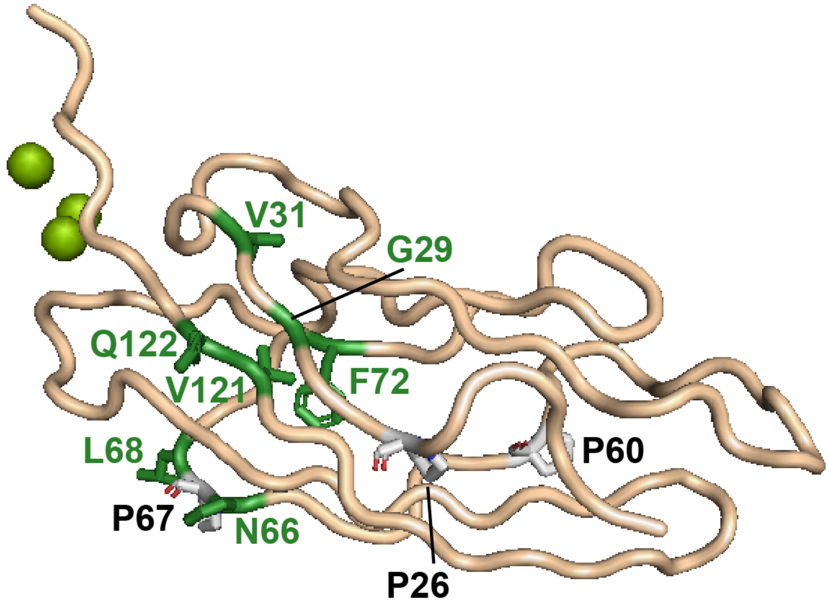

Figure S1: Conformational variability. The 7 residues showing doublets or elongated signals cluster in loop and ⊚-regions near the Ca^{++} end of EC1, and particularly near cisPro67.
